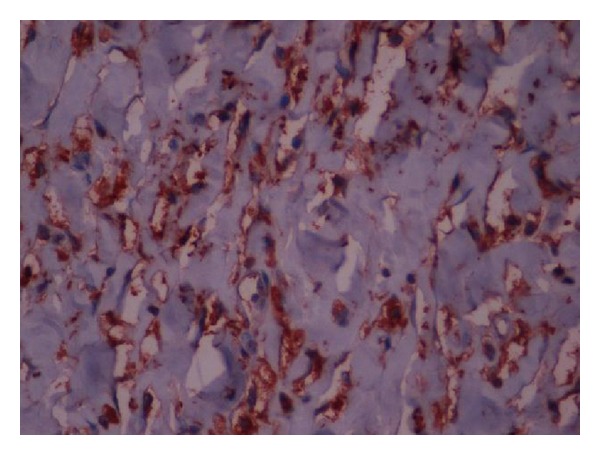# Extragastrointestinal Stromal Tumor during Pregnacy

**DOI:** 10.1155/2012/846747

**Published:** 2012-10-18

**Authors:** Ilay Gözükara, T. U. Kutlu Dilek, Hüseyin Durukan, Duygu Düsmez Apa, Suna Kabil Kucur, Saffet Dilek

**Affiliations:** ^1^Obstetric and Gynecology Department, Erzurum Nenehatun Obstetric and Gynecology Hospital, Erzurum, Turkey; ^2^Obstetric and Gynecology Department, Medical Faculty Hospital, Mersin University, Mersin, Turkey; ^3^Pathology Department, Medical Faculty Hospital, Mersin University, Mersin, Turkey

## Abstract

Extragastrointestinal stromal tumors (EGISTs) are mesenchymal neoplasms without connection to the gastrointestinal tract. Gastrointestinal stromal tumors (GISTs) and EGIST are similar according to their clinicopathologic and histomorphologic features. Both of them most often express immunoreactivity for CD-117, a c-kit proto-oncogene protein. The coexistence of GIST and pregnancy is very rare, with only two cases reported in the literature. In this paper, we presented the first EGIST case during pregnancy in the literature.

## 1. Introduction

 Gastrointestinal stromal tumors are rare visceral neoplasms that arise from gastrointestinal tract and most often express immunoreactivity for CD-117, a c-kit proto-oncogene protein [1]. The clinicopathological and immunohistochemical features of CD-117 positive mesenchymal tumors without connection to the gastrointestinal tract, are known as extragastrointestinal stromal tumors [2]. The coexistence of GIST and pregnancy is very rare, with only two cases reported in the literature [3]. 

In this study, we presented the first EGIST case report during pregnancy in the literature. 

## 2. Case

A 21-year-old woman in her fifteenth week of pregnancy presented to the emergency department with abdominal pain. Her past medical history was negative for any disease. On physical examination, abdominal mass was found reaching to the umbilicus level. Obstetric assessment including ultrasonography determined a viable intrauterine pregnancy with normal anatomical findings. Pelvic mass was primarily considered as a leiomyoma uteri. She underwent a median laparotomy that demonstrated a mass lobulated, white, smooth surface with 17 × 17 × 10 cm in diameter originated from omentum. Frozen section analysis was done intraoperatively, and the specimen was evaluated as a benign mesenchymal tumor. The final histological diagnosis of the resected tumor confirmed the existence of EGIST. Immunohistochemical analysis revealed strong c-kit immunoreactivity and positive reactions for CD-34 and desmin.

## 3. Pathology 

**Figure 1:**
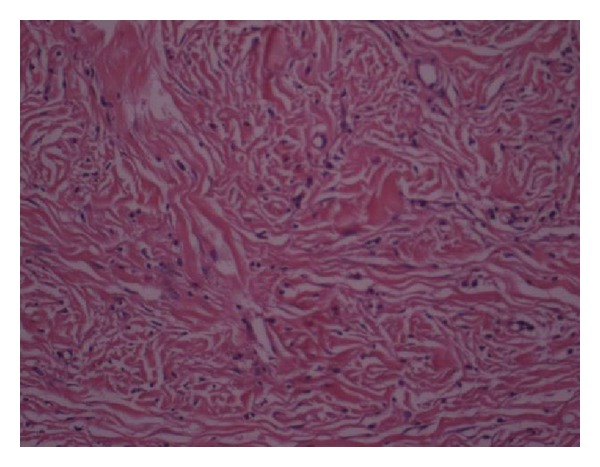


Histomorphological examination revealed spindle cell proliferation in a matrix of fibrillary collagen. There was marked intratumoral inflammatory cell infiltration. No necrosis, cellularity, and mitotic activity were noted (Figure 1). In immunohistochemical examination, there were strong CD-17(c-kit) and weak CD34 expression (Figure 2).

## 4. Discussion

GIST are the most common mesenchymal tumors of the GIS, with approximately 70% occurring in stomach, 20%–30% occurring in the small intestine, and less than 10% in the esophagus, colon, and rectum. Rare cases are identified outside the GI tract and are collectively known as EGIST [4]. Most of EGISTs are found in the omentum, but also it was identified in the scrotum, bladder, pharynx, inguinal hernia, hypochondrium, pancreas, pelvis, retroperitoneum, and rectovaginal septum [4, 5]. In 2000, Reith et al. termed these tumors as extragastrointestinal stromal tumors (EGISTs), which account for less than 10% of all stromal tumors of the abdomen [6].

There is not any EGIST case reported together with pregnancy in the literature. This is the first report of it. 

 GIST and EGIST are similar according to their clinicopathologic and histomorphologic features [3]. Tumors may appear as well-defined or polylobulated firm mass with or without cystic, necrotic degeneration. They range in size from 2.1 to 32 cm with most of the tumor more than 5 cm in size as they have enough space to grow and are usually inseparable from the wall of the stomach or intestine [1, 7, 8]. EGISTs usually affect females whose ages range from 31 to 82 years (mean, 58 years). Abdominal ultrasonography is the first-line screening modality to detect the presence of a mass and to differentiate cystic from solid tumors. But it usually cannot identify the primary site of the tumor and its characteristics. CT and PET scans can identify the primary tumor site and relationship with other organs. Because of pregnancy, only ultrasonography evaluation was used in this case [8].

 The tumors are composed of either purely rounded epithelioid cells (predominantly) or short fusiform cells in a fine fibrillary collagenous background. Rarely, mixed pattern is also encountered. Similar to GISTs, EGISTs also display varying amounts of stromal degeneration (hyalinization, myxoid change and cyst formation). However, skeinoid fibers, a common marker in GIST of the small bowel, are absent in this tumor. In this case spindle cell proliferation in fibrillary collagen was observed but there was no other necrotic or cellular changes [6].

 There are usually mutations in either KIT-CD117 or PDGF (platelet-derived growth factor) with these kind of mesenchymal tumors [9]. 95% of GISTs are found with CD117 somatic mutation. CD117 is a tyrosine kinase transmembrane receptor located on chromosome 4 which is also the product of proto-oncogene c-kit [1]. 

 EGIS tumor diagnosis is confirmed with positive and diffuse, strong immunostaining for c-kit (CD117) [9]. EGISTs are 7% of CD117 positive staining abdominal mesenchymal tumors [3]. In this case there were strong CD-17(c-kit) and weak CD34 expression.

 Pathological diagnosis is pivotal for the postoperative therapy due to unique treatment with single agent KIT inhibitor imaitinib mesylate [9]. Definitive treatment is complete surgical excision for the localized diseases. In the present case surgical treatment without any necessity for chemotherapy was done.

## Figures and Tables

**Figure 2:**